# Genetic Variation and Atherosclerosis

**DOI:** 10.2174/138920208783884856

**Published:** 2008-03

**Authors:** Erik Biros, Mirko Karan, Jonathan Golledge

**Affiliations:** Vascular Biology Unit, School of Medicine, James Cook University, Townsville, QLD 4811, Australia

**Keywords:** Atherosclerosis, genetic polymorphism, risk factors.

## Abstract

A family history of atherosclerosis is independently associated with an increased incidence of cardiovascular events. The genetic factors underlying the importance of inheritance in atherosclerosis are starting to be understood. Genetic variation, such as mutations or common polymorphisms has been shown to be involved in modulation of a range of risk factors, such as plasma lipoprotein levels, inflammation and vascular calcification. This review presents examples of present studies of the role of genetic polymorphism in atherosclerosis.

## INTRODUCTION

Atherosclerosis and the complications of this disease are the leading cause of disability and death in Western societies. The development of advanced atherosclerosis is a slow progressive process that starts in childhood and remains asymptomatic for many decades, with complications such as myocardial infarction, stroke or peripheral ischaemia usually occurring in later life [1]. Recognised risk factors for the development of atherosclerosis include hypertension, diabetes, dyslipidaemia, obesity, smoking, ageing and sedentary life style [2, 3]. Twin studies have suggested that the heritability of coronary atherosclerosis based on fatal cardiac events is between 38 and 57% [4, 5]. Epidemiology studies suggest that a positive family history is independently associated with the incidence of cardiovascular events [6]. Besides major genetic determinants of traditional cardiovascular risk factors, such as those influencing the lipid profile, it is likely that a large number of additional genetic factors interact with environmental factors to determine overall cardiovascular risk. Development of molecular biology techniques has promoted identification of these candidate genes by using high-throughput technologies, such as genome-wide scans. This review provides examples of advances in our understanding of the role of the genomic variations in atherosclerosis and candidate genes which have been related to atherosclerosis.

## CONSIDERATIONS IN GENOME STUDIES

The methods used to examine genetic factors involved in chronic diseases such as atherosclerosis are evolving rapidly. Traditionally studies have centred on examining sub-population where the early onset of presentation, presence of multiple affected family members and mode of inheritance suggest a very important role for genetics in the development of the disease. Using techniques such as affected-relative pair linkage analysis it is possible to identify genomic regions in which a single gene involved in the disease may be located. In most chronic diseases however it is likely that multiple genes acting in concert under the influence of a range of environmental factors determine development of diseases, such as atherosclerosis. The identification of these genes has mainly followed a candidate gene approach. Based on understanding of the pathogenesis of atherosclerosis genes involved in control lipid mobilisation, inflammation and endothelial function for example have been examined for association with atheroma presence or cardiovascular events. The selection of which sites of genetic variation within the genes to examine is complex depending on frequency of the variation, sample size or power, estimated functional effect and ability to predict other related genetic polymorphisms [7]. Over the last few years with the rapid advances in genotyping there has been the introduction of genome wide association studies. This approach uses a screening rationale to examine up to 1 million allelic variations in large numbers of samples from cases and controls. The approach generates enormous data set which will ultimately enable analysis of epigenetic interaction and environmental responsive loci. At present our ability to analyse such data sets is relatively rudimentary.

In this review we will mainly discuss examples of the candidate gene approach since this has been the main technique applied. The selection of genes has been primarily based on our present understanding of the biology of atherosclerosis from *in vitro*, animal and pathological studies. An important issue in association studies is whether the correct allelic variation site in the gene of interest has been examined. Given the enormous number of variation even within a single gene it is usually not possible to examine every one. The size of cohort required for sufficient power to detect likely influence of a single genetic variation is very dependant on the frequency of the minor allele within the population being studied. For polymorphisms imparting a relative risk of 1.5 or less minor allele frequencies >5% are required for most achievable samples sizes [8]. Thus it is usual to target variations which are more common. Since a number of allelic variations are inherited together another important way of minimising the number of variations genotyped is by selected those which are maximally linked to other common sequence changes. It has been demonstrated that the human genome can be organized into haplotype blocks i.e. regions of strong linkage disequilibrium (LD) separated by regions of low level LD [9-13]. The availability of detailed linkage information on different populations, such as that presented in HapMap (The International HapMap Consortium 2007 [14]), allows researchers to select tagging single nucleotide polymorphisms and assesses different combinations of polymorphisms or haplotypes. Another consideration in the selection of the allelic variations to be examined is its functional effect. Databases, such as PANTHER (Protein ANalysis THrough Evolutionary Relationships) gene ontology database [15] can be utilised to provide estimates of the functional effects of allelic variations. PANTHER uses subSPEC (substitution position-specific evolutionary conservation) score to predict the probability (P_deleterious_) that a given coding variant will cause a deleterious functional change, such that a subPSEC score of -3 corresponds to a P_deleterious_ of 0.5 [16, 17].

A number of issues are raised in the selection of cases and controls for association studies, such as what criteria are used to define cases (e.g. history, documented events, imaging), are controls from the same populations and has atherosclerosis been excluded thoroughly (history or imaging) [18].

## GENETIC VARIANTS IN LIPOPROTEIN METABOLISM

Aberrations in lipid handling are one of the fundamental mechanisms that have been linked to atherosclerosis, particularly in patients presenting with positive family history and at younger age [19]. About three quarters of low density lipoprotein (LDL) in the blood stream is taken up by hepatic or peripheral cells *via *LDL receptors (LDLR) on the cell surface. These receptors recognize apolipoprotein B and apolipoprotein E with high affinity. In hepatocytes, the absorbed LDL is reused for lipoprotein synthesis and excess cholesterol is secreted into bile. In non-hepatic cells, the absorbed LDL supplies the cholesterol content essential for cell function.

There is also reverse transport of excess cholesterol from the periphery back to the liver by high density lipoprotein (HDL) secreted by liver as nascent pre β-HDL. Cholesterol efflux into mature HDL (HDL-C) is mediated by the ATP-binding cassette (ABC) transporter family of membrane proteins, especially ABCA1. HDL-C can then return to the liver *via *a hepatocyte scavenger receptor (SCARB1). In the liver, cholesterol can be stored within hepatocytes, converted into bile acids by the cytochrome P450 member CYP7A1 (7-α-hydroxylase), or transported directly by ABCG5 and ABCG8 transporters. There is accumulated evidence that impaired homeostasis in these processes has a strong genetic background [20, 21]. 

### Low-Density Lipoprotein Receptor Gene (LDLR)

Familial hypercholesterolemia (FH) is a monogenic autosomal codominant trait affecting 1 in 500 individuals in Caucasian populations [19] with an estimated world prevalence of about 0.2% [22]. FH is characterized by elevated plasma cholesterol bound to LDL (LDL-C) due to a deficiency in LDLR activity on the cell surfaces [23].

*LDLR* is located on chromosome 19 [24] at 19p13.1-p13.3 [23] and encodes a protein of 860 amino acids [25]. There are more than 800 different allelic variations in this gene described [26-30], which have been divided into 5 classes according to the effect they have on LDLR function [26]. Class 1 mutations lead to an inability to produce immunoprecipitable protein (receptor-negative mutations), whereas classes 2-5 affect function of the receptor (receptor-defective mutations). FH heterozygotes express half the normal number of functional LDLR on their cell surfaces leading to a twofold elevation in circulating LDL-C concentration (300-500 mg/dL) associated with premature atherosclerosis development [19]. The rare FH homozygotes (1 in 1,000,000) express only few or no functional receptors on their cell surfaces. These individuals have plasma LDL-C of 600-1,200 mg/dL and may suffer fatal heart attacks before the age of 20 (reviewed in [19]).

**Fig. (1):**
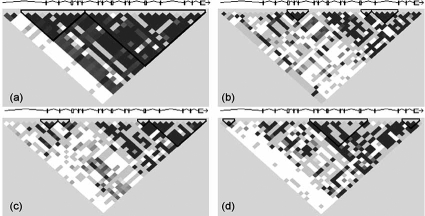
Complex correlation structure to the HapMap genotype data at *LDLR* gene locus (19p13.2) in four populations. (**a**) Utah residents with ancestry from northern and western Europe, (**b**) Han Chinese in Beijing (China), (**c**) Japanese in Tokyo (Japan), (**d**) Yoruba in Ibadan (Nigeria).

There is promising evidence that DNA polymorphisms within *LDLR* could be useful to monitor the inheritance of FH. Most association studies confirm the link between the restriction fragment length polymorphisms (RFLPs) in *LDLR* exon 12 or 13 and FH [31-34]. Two research groups reported strong linkage disequilibrium of two microsatellite markers (D19S394 and D19S221) to the most common *LDLR* mutations (p.C152R, p.S265R, p.V408M and p.G528D), accounting for 20% and 60% of FH heterozygotes, respectively [35]; [36]. On the other hand, there is some consideration to generalize these polymorphisms as molecular markers for FH. At least, the data are based on individuals of different ethnic origin. The comparisons of haplotypes found in subjects of diverse ethnicity suggest heterogeneity among populations. For example, by using the *LDLR* HapMap genotype data [14], there were found distinct haplotype block patterns in four different populations (Fig. **1**). To monitor the inheritance of FH by using the common *LDLR* genetic variations, it is necessary to establish the panel of informative polymorphisms for any population of interest.

### Apolipoprotein B Gene (APOB)

Human *APOB* is located on the short arm of chromosome 2 [37] at 2p24-p23 [38] and codes for the main apolipoprotein of chylomicrons and LDL. The protein occurs in plasma in two main isoforms *via *a unique mRNA editing process: intestinal apoB-48 and hepatic apoB-100. ApoB-48 is identical to the N-terminal 48% of full-length apoB-100 [39, 40]. Two genetic disorders, familial hypobetalipoproteinemia (FHBL) and familial ligand-defective apoB-100 (FDB) are attributable to mutations in the *APOB* gene. FHBL is an autosomal dominant trait characterized by low plasma levels of total cholesterol (TC), LDL-C, and apoB. FDB is an autosomal dominant disorder accompanied by hypercholesterolemia and premature atherosclerosis [41]. Mutations within *APOB* cause the production of truncated proteins (between apoB-4.6 to apoB-89) and can be responsible for FHBL (reviewed in [42]). Defects in the C-terminus of the apoB LDLR-binding domain are accountable for FDB, which is clinically indistinguishable from FH [43].

The amino acid substitution p.R3500Q has been found to be strongly associated with hypercholesterolemia and FDB [41-46]. The frequency of p.R3500Q in Europe is about 0.09% [47, 48]. The sequence variation p.R3531C impairs LDLR binding capacity up to 49% of normal and increases LDL-C concentration in plasma [49]. The p.R3480W substitution leads to impaired binding to LDLR [50].

*APOB* is highly polymorphic gene with more than 80 allelic variations within its entire sequence and other common genetic variants. For example, the APOB signal peptide exhibits variability in length (24 or 27 amino acids) due to the insertion (*ins* allele) or deletion (*del* allele) of three codons [51-53]. The frequency of *del* allele is about 30% in Caucasians [54] and has been associated with altered plasma total cholesterol and LDL-C levels in different ethnic groups [55-60] but interestingly not with the risk of vascular diseases [54]. Thus variation at the *apoB* gene may act in pathogenesis of vascular diseases through mechanisms not directly related to effects on measured lipid traits. Linkage analysis of quantitative trait loci (QTL) associated with increased plasma levels of apoB protein provided evidence for chromosomal segments at 1p21-31 and 17p11-q21 with LOD scores 2.2 and 3.7, respectively [61] in familial combined hyperlipidemia (FCHL) patients. While linkage peak on chromosome 1 fits to the dietary energy and nutrient intake linkage peak [62]; the peak at chromosome 17 corelates to QTL for type 2 diabetes [63] and blood pressure variation and hypertension [64, 65]; all risk factors for cardiovascular diseases.

Several restriction fragment length polymorphisms (RFLP) and variable number of tandem repeats (VNTR) polymorphisms have also been described within *APOB* coding as well as 3’-untranslated region (3’-UTR), [66, 67]. Studies examining the association of these common genetic variations in *APOB* with atherosclerosis have led to inconsistent findings [68-72].

### Apolipoprotein E Gene (APOE)

APOE is a key protein of the lipid-transporting system [73, 74], regulating serum cholesterol [75], and participating in formation of high density lipoprotein (HDL) particles [76]. Hepatic parenchymal cells mediate production of all peripheral APOE [73]. APOE-containing particles are rapidly removed from the circulation by binding to LDLR or LDLR-like protein receptor (LRP)-mediated endocytosis in the liver [77-79].

*APOE* is mapped to chromosome 19 at 19q13.2 [80]; [81] where it is linked to apolipoprotein C1 and C2 [82]. *APOE* has four exons and three introns [83]. The gene is polymorphic with three major alleles (*APOE2*, *APOE3*, and *APOE4*) translating into three protein isoforms ε2, ε3, and ε4 [84-86]. They differ from each other only by single amino acid substitutions at residues 112 and 158 [87, 88]. The most common variant is ε3 (~78% in Caucasians; [89]) and carries cysteine and arginine at position 112 and 158, respectively. Minor molecular change has profound pathological consequences. While *E3* allele codes for wild-type protein (Cys^112^/Arg^158^), *E2* (Cys^112^/Cys^158^) is associated with type III hyperlipoproteinemia (HPL III), and allele *E4* (Arg^112^/Arg^158^) has been implicated in atherosclerosis [90, 91]. 

Other polymorphisms like g.491A>T, g.427C>T, g.219G>T found within the *APOE* promoter [92, 93] and coding g.113G>C [94] regions have been linked with atherosclerosis. Regarding the *APOE *promoter g.491A>T SNP, circulating APOE concentrations are elevated in TT homozygotes compared to individuals bearing AA genotype [95]. The *APOE* promoter g.219G>T and coding g.113G>C allelic variations have been associated with changes in LDL-C and total cholesterol (TC) concentrations [96]. Present evidence suggests that the influence of other *APOE* genetic variants is small by comparison to its major alleles (*E2*, *E3*, and *E4*).

### ATP Binding Cassette Transporter 1 Gene (ABCA1)

ABCA1 functions as a cholesterol efflux pump in the apolipoprotein mediated cellular lipid removal pathway [97]. Specifically, ABCA1 mediates the transport of cholesterol from cells to lipid-poor apolipoprotein (apo) A-I, the major HDL protein (reviewed in [98]); [99]. Mutations within the *ABCA1* gene explained the molecular basis of Tangier disease, the autosomal codominant trait characterized by reduced serum HDL (<5mg/dL) and subsequent premature coronary atherosclerosis [100-102]. Disease causative mutations (e.g. g.3283_3284delTC in exon 22; [103]) have led to the hypothesis that common polymorphisms in *ABCA1* control serum lipoprotein levels as a risk factor of atherosclerosis in the general population. *ABCA1 *is mapped to chromosome 9 at 9q31.1 [104] and displays multiple common variations within its sequence. Some of these polymorphisms correlate to circulating lipid or lipoprotein levels [105-110].

Among major lipid-relevant genes, *ABCA1* haplotypes influence HDL and LDL/HDL ratio by only 10% and 4%, respectively [111]. Thus, it is not surprising that other studies by Zwarts *et al*. [108] and Tregouet *et al*. [112] (ECTIM - Etude Cas-T´emoins de l’Infarctus du Myocarde study) reported that *ABCA1* polymorphisms are associated with the frequency and severity of coronary events independent of changes in the circulating lipoprotein levels. Promoter as well as 5’-UTR variants have been associated with multiple increases in coronary events and severity of coronary artery disease.

### CYP7A1 Gene

This gene codes for a member of the cytochrome P450 superfamily of enzymes and is located on chromosome 8 at 8q11-q12 [113]. *CYP7A1* is one of the critical genes playing a role in the classical pathway of cholesterol conversion into bile acids (reviewed in [114]). Genetic variations in *CYP7A1* have been extensively studied and associated with hypercholesterolaemia in some [115-118] but not all studies [119, 120]. When association was reported, it consistently related to the promoter region of the gene. There is not any common non-synonymous polymorphism within its coding sequences, except the exon 3 c.762A>G with the estimated frequency of heterozygosity at about 0.006. While it is not known any functional variants of this polymorphism, it can not be excluded that there is another close-linked gene with common polymorphisms that is involved in the control of plasma lipids. Gene layout around the *CYP7A1* locus indicates for syntenin (SDCBP), the gene coding for protein with pleiotropic functions, including the cell signalling and lipid metabolism.

### ABCG5/ABCG8 Locus

ATP-binding cassette sub-family G members 5 and 8 (*ABCG5* and *ABCG8*) are tandemly arranged in a head-to-head orientation on chromosome 2p21 [121, 122]. The ABC transporters, ABCG5 (sterolin-1) and ABCG8 (sterolin-2) function in trafficking of all sterols, including cholesterol [123]. Multiple mutations in either *ABCG5* or *ABCG8* may result in sitosterolaemia [124-127]; a rare autosomal recessive trait characterized by increased intestinal absorption and decreased biliary excretion of sterols. Subjects with this disorder develop premature coronary atherosclerosis [128]. Genetic polymorphisms within the sitosterolaemia locus explain inter-individual differences in sterol metabolism and plasma sitosterol [129]. Polymorphic variants p.Q604E (ABCG5), p.D19H and p.T400K (ABCG8) have been associated with plasma plant sterols and lipid levels in normocholesterolaemic individuals or mildly hypercholesterolaemic patients [130-133]. Variant p.M429V (ABCG8) was linked to higher cholesterol absorption efficiency in hypercholesterolaemic patients [134]. Pandit *et al*. [135] published a detailed haplotype map of the sitosterolaemia locus. They found *ABCG8* gene to be more polymorphic than *ABCG5*. Some of allelic variations were in strong LD and very unique to different ethnic groups. A polymorphism in *ABCG8* has been linked to the response of serum LDL cholesterol to atorvastatin therapy [136]. This is an example of how genetic variability can influence the outcome of treatment of atherosclerosis not just its development.

### SCARB1 Gene

Scavenger receptors (SRs) are receptors for modified forms of lipoproteins including oxidized and acetylated LDL (ox-LDL and ac-LDL), lipopolysaccharides (LPS) of Gram-negative bacteria, and other poly-anionic ligands [137, 138]. They are divided into eight classes (A-H) and the majority of them are expressed on the surface of antigen presenting cells – APC [139]. Several informative sequence variations have been found within the member of the scavenger receptors gene family B, scavenger receptor class B type 1 gene (*SCARB1*). This gene spans approximately 75kb on chromosome 12 at 12q24.31 [140, 141]. Allelic variations within the coding sequence of the *SCARB1 *gene have been found in exons 1 and 8 [142, 143]. Both polymorphisms exhibit anti-atherogenic properties and the phenotype seems to vary with gender. While exon 1 variant was associated with increased HDL-C and lower LDL-C in men, exon 8 variation was linked to lower LDL-C concentrations in women [142, 144]. Exon 8 allelic variation has no impact on amino acid sequence and is believed to be in linkage disequilibrium with other functional mutations within *SCARB1* or adjacent loci at 12q24 chromosomal region. Other putative susceptibility genes in this region include TP53 regulated inhibitor of apoptosis 1 (*TRIAP1*) and ATP-binding cassette sub-family B member 9 (*ABCB9*).

### Other Loci Controlling Lipid Metabolism

Several genome-wide linkage scans have been performed to detect quantitative trait loci (QTLs) regulating lipid or lipoprotein metabolism. As the most significant results, these studies identified linkage between 19p13 (LOD score 3.00) and plasma cholesterol concentration [145]. Chromosomes 16q23.1-24.2 (LOD score 3.73), [146], 12q14.1 (LOD score 4.06), [147] and 15q21 (LOD score 4.77), [148] were linked to HDL-C. While sequences at 1q43, 11q23.2, 15q25.1 and 19q13.32 (LOD scores 2.50, 3.22, 3.11 and 3.59), [147] and 1p33-35 (LOD score 3.60), [149] were linked with LDL-C. Chromosomal segments at 15q12-q13.1 (LOD score 3.88), [150] and 7q36 (LOD score 2.98), [151] linked to TG.

Several prominent candidate genes residing within these linkage regions are shown in Table **1**. Among them, mutations in proprotein convertase subtilisin/kexin type 9 (*PCSK9*) gene and low density lipoprotein receptor adaptor protein (*LDLRAP1*) gene (encoding a protein required for clathrin-mediated internalization of the LDL receptor by liver cells) have already been reported to affect LDLR pathway. Recent data indicates that about ten *PCSK9* mutations are associated with hypercholesterolemic or hypocholesterolemic phenotype [152-156]. Recessive null mutations within *LDLRAP1* were observed to co-segregate with hypercholesterolemia [157] that is particularly common in Sardinia [158], presumably because of the founder effect.

## BEYOND LIPOPROTEINS

An abundance of data has shown the relationship between circulating lipoprotein variables and risk of cardiovascular events. On the other hand, half of all coronary events occur in subjects with below-average cholesterol levels [3]. In this section we discuss some other genes that have been associated with atherosclerosis. Inflammation plays an important role in the development and complications of atherosclerosis (reviewed in [2]); [159]. Monocyte-macrophage recruitment appears to be particularly important. This process engages multiple genes. As an example we highlight transforming growth factor beta 1 (*TGFB1*) and scavenger receptors or toll like receptor family (*TLR*). Calcification is another important feature of advanced atherosclerosis. We also highlight two genes linked to calcification i.e. secreted phosphoprotein 1 (*SPP1*), also known as osteopontin (*OPN*) and tumor necrosis factor receptor superfamily member 11B (*TNFRSF11B*), also known as osteoprotegerin (*OPG*). Matrix metalloproteinases have been identified in many important stages in atheroma formation including inflammation and plaque rupture [160].

### Transforming Growth Factor Beta 1 Gene (TGFB1)

This gene is located on chromosome 19 at 19q13.1-q13.3 [161, 162] and codes for a pleiotropic cytokine, which regulates proliferation and differentiation of a wide variety of cell types [163]. The quantitative production of TGFB1 differs amongst individuals due to genetic polymorphism with estimated heritability of approximately 0.54 [164]. Increased plasma levels of the cytokine are associated with the T-allele of the promoter g.509C>T sequence variation due to loss of negative regulation by the complex of transcription factors AP1, JUND, and c-FOS [165]. This complex can bind TGFβ1 only when wild-type C-allele is present. The deregulation of this pathway has been suggested to play a role in mediating predisposition to various diseases, including atherosclerosis [166-169]. For example a polymorphism in *TGFβ1* has been associated with stroke [170].

### Toll Like Receptors (TLR) and Scavenger Receptors (SR) 

Toll like receptor family contains the pattern recognition receptors (PRRs) of various pathogen-associated molecular patterns (PAMPS), [171]. There are more than ten human members of the family specialized in recognition of both endogenous and exogenous ligands [172-174]; (reviewed in [175]). Ligation of TLR4 that signals through myeloid differentiation primary response protein MyD88-dependent pathway towards the pro-inflammatory nuclear factor κB (NF-κB) signalling cascade (reviewed in [176]) has been proposed to play an important role in the initiation and progression of atherosclerosis (reviewed in [177]). TLR4 has been demonstrated in macrophages within atherosclerotic plaques and shown to be up-regulated by ox-LDL [178]. Hoebe *et al*. [179] demonstrated that TLRs and macrophage type A scavenger receptor (SR-A) can functionally cooperate in macrophage-bacteria interactions and signalling. Recent work by Seimon *et al*. [180] has revealed that macrophages in the atherosclerotic plaques apoptose when *TLR4* and *SR-A* are activated at the same time. SR-A ligands trigger macrophage apoptosis *via *endoplasmic reticulum (ER)-stressed pathway by redirection of TLR4 signalling from pro-survival to pro-apoptotic. This combination of signalling can lead to plaque rupture and arterial thrombosis.

Given the role of TLRs and SRs in cell signalling, identification and functional characterization of polymorphisms in genes coding for these receptors may influences important stages of the development and complications of atherosclerosis. Common, co-segregating missense coding allelic variations in the *TLR4* gene on chromosome 9q32-q33 [181] encode the p.D299G, and p.T399I substitutions that blunt receptor signalling [182]. A link between hypo-responsive TLR4 and susceptibility to cardiovascular events has been assessed in multiple association studies with variable observations. Ameziane *et al*. [183], Edfeldt *et al*. [184] and Holloway *et al*. [185] reported the association of TLR4 allelic variation with myocardial infarction; however other studies could not confirm these findings [186, 187]. These inconsistencies may be clarified by simultaneous examination of sequence variations with other polymorphic associates of the TLR4 physiological pathway.

The class A scavenger receptors are responsible for approximately 75% of the degradation of ox-LDL and ac-LDL [188]. Matsumoto *et al*. and [189] Emi *et al*. [190] mapped the *SR-A* gene on chromosome 8 to 8p22. Besides several germ-line mutations identified within this gene [191], there are more than 200 polymorphisms mapped to the entire genomic region of the *SR-A *including three putative non-synonymous sequence variations in exon 4 at amino acid position 105 (rs13306549), exon 5 at amino acid position 269 (rs13306543) and exon 6 at amino acid position 275 (rs3747531).

### Secreted Phosphoprotein Gene (SPP1)

SPP1 is an acidic glycophosphoprotein normally found in mineralized tissues acting as an inhibitor of apatite crystal growth [192]. SPP1 has been demonstrated at sites of calcification in atherosclerotic plaques and in calcified aortic valves [193-195]. SPP1 has been implicated in a variety of mechanisms important in atherosclerosis including proliferation and migration of endothelial cells, macrophages, and vascular smooth muscle cells [196, 197].

The gene coding for SPP1 was assigned to chromosome 4q21-q25 [198]. There are more than sixty allelic variations at the *SPP1* locus, including six non-synonymous variants coding for an amino acid change. Recently, Taylor *et al*. [199] in the CARDIA study and Brenner *et al*. [200] reported an *SPP1* allele specific association with coronary artery calcification and stroke, respectively.

### Tumor Necrosis Factor Receptor Superfamily Member 11b Gene (TNFRSF11B)

*TNFRSF11B* gene was mapped to chromosome 8q24 [201]. TNFRSF11B belongs to the tumour necrosis factor (TNF) receptor super-family and acts as a decoy receptor of the receptor activator of the NF-κB ligand (RANKL) that is a strong inducer of osteoclast differentiation acting through its receptor activator of NF-κB (RANK) receptor [202].

Recent studies on TNFRSF11B tissue expression, serum levels, or gene polymorphisms also suggest an important role of the RANKL/RANK/TNFRSF11B cytokine system in atherosclerosis and vascular calcification. While TNFRSF11B is detected in both normal and atherosclerotic tissue, RANKL/RANK is expressed only in calcified arteries [203]. Moreover, TNFRSF11B is more abundant in symptomatic than in asymptomatic carotid plaques, suggesting a role in the plaque instability [204].

Recent study of four promoter polymorphisms (g.163A>G, g.209G>A, g.245T>G and g.950T>C) in the promoter region of the *TNFRSF11B* gene in a Korean cohort failed to identify any association with coronary artery disease (CAD) or aortic calcification [205]. On the other hand, previous haplotype analysis of the g.950T>C and g.G1181G>C (exon 1) showed significant association with CAD in Caucasian men [206] suggesting ethnic differences. An increased risk of CAD was reported in carriers of at least one C allele of both polymorphisms. The C allele at position 950 also correlated with serum TNFRSF11B levels.

### Matrix Metalloproteinases (MMPs)

MMPs, a family of diverse enzymes consisting of 24 zinc-dependent endopeptidases, process various components of the extra-cellular matrix and cell surface proteins (reviewed in [207]). Altered MMP activities have been implicated in a variety of pathological processes, including atherosclerosis [208]. Several members of the *MMP* family have functional polymorphisms that have been assessed for association to atherosclerosis, e.g. *MMP1*, *MMP3*, and *MMP9*. For these genes, promoter polymorphisms have been linked to increased risk of carotid artery stenosis [209, 210].

### Other Candidate Genes

A genome-wide scan by Lange *et al*. [211] revealed two distinct linkage peaks at chromosomal regions 6p21.3 (LOD score 2.22) and 10q21.3 (LOD score 3.24), which may harbour genes associated with coronary atherosclerosis. Suggested candidate genes within these regions include collagen type XI α2 (*COL11A2*) and allograft inflammatory factor 1 (*AIF1*) on chromosome 6, plus collagen type XIII α1 (*COL13A1*) and bone morphogenetic protein receptor type 1A (*BMPR1A*) on chromosome 10. *BMPR1A* was found overexpressed in asymmetric dimethylarginine (ADMA) conditioned coronary artery endothelial cells under pathophysiological concentrations [212]. ADMA is a naturally occurring component of plasma that inhibits nitric oxide synthesis and was linked to major adverse cardiovascular events or death [213].

Association of coronary artery disease with polymorphism on chromosome 6 has been confirmed by another study reporting a linkage peak between 6p12–p22 [214]. The investigators suggested vascular endothelial growth factor (*VEGF*) localized at 6p12 (LOD score 2.21) as a promising candidate gene. A number of other studies have suggested genomic sites linked with coronary atherosclerosis and/or myocardial infarction [215-223]. Helgadottir *et al*. [220] in their linkage study of 713 cardiac patients reported linkage peak at 12q22 coding for leukotriene A4 hydrolase (*LTA4H*) as a candidate gene. The authors proposed the role of leukotrienes in mediating individual susceptibility to myocardial infarction. Table **2** provides examples of putative genes residing within these loci and their amino acid substitution causing by non-synonymous SNPs. We scored these polymorphisms to predict their functional significance (subPSEC value) by using the PANTHER’s coding SNP analysis tool [17]. The substitutions T600S (*LTA4H*) and R443C (*BMPR1A*) showed the highest subPSEC values of -2.62 and -8.29 with probability of deleterious effect (P_deleterious_) of 0.41 and 0.99, respectively; suggesting these variants for further studies in relation to cardiovascular diseases.

## CONCLUSION

Atherosclerosis is a complex disorder depending on an interaction between genotype and environment. The relative contribution of genes and environment varies from one patient to another. There is no unifying genetic pattern that is associated with atherosclerosis. Mendelian disorders, such as FH or sitosterolaemia explain only a small part of disease risk indicating the involvement of complex non-Mendelian traits and their combined effects. While some individuals suffer from hereditary impaired lipoprotein homeostasis, for others, chronic inflammation or vascular calcification may be the prevalent cause of their increased susceptibility. Large studies examining different populations are on going and will shed further light on the importance of different genes for different presentations of atherosclerosis.

## Figures and Tables

**Table 1.** Candidate Genes within Chromosomal Linkage Regions Involved in Lipoprotein Metabolism

Ref.	Subjects	Ethnicity	Linkage Site	LOD	Related Phenotype	Candidate Gene(s)
[147]	Obese families	Caucasian	12q14.1	4.06	HDL-C	*SOAT2, APOF, CYP27B1*
[224]	Population based study	Caucasian	5p13.1	3.64	HDL-C	
[146]	Population based study	Mexican Americans	16q23.1-24.2	3.73	HDL-C	*LCAT*
[225]	Familial combined hyperlipidemia	Caucasian	3p14	3.00	HDL-C	*ACOX2*
[148]	Population based study	Caucasian	15q21	4.77	HDL-C	*LIPC*
[226]	Low HDL-C families	Caucasian	8q23	4.70	HDL-C	
[147]	Obese families	Caucasian	1q43 11q23.2 15q25.1 19q13.32	2.50 3.22 3.11 3.59	LDL-C	*ABCB10*, *GGPS1* *ACAT1*, *APOA1*, *APOC3* *CYP11A* *LRP3*, *APOE*,*LIPE*,*APOC1*
[227]	Type 2 diabetes families	Caucasian	3p25 2p23 19p13	2.47 2.17 2.23	LDL-C	*PPARG* *APOB*, *LPIN1*, *ABCG5*, *ABCG8* *LDLR*
[149]	Healthy children	African Americans	1p33-35	3.60	LDL-C	*PCSK9, LDLRAP1, CYP4A11*
[147]	Obese families	Caucasian	2p14 11p13 11q24.1	1.75	TG	*FABP1* *ABCC8*, *LRP4* *ACAT1*, *APOA1*, *APOC3*, *ACAD8*
[228]	Hypertriglyceridemic families	Caucasian	15q21-q24	2.56	TG	*LIPC*
[151]	Obese families	Caucasian	7q36	2.98	TG	*INSIG1, ABCF2, FABP5L3*
[150]	Type 2 diabetes families	Caucasian	15q12-q13.1	3.88	TG	*LIPC*
[229]	Population based study	Caucasian	7q32.3-qter	2.50	TG/HDL-C ratio	*ABCF2*
[145]	Type 2 diabetes families	Pima Indians	19p13	3.00	TC	*INSR, C3*

*SOAT2* sterol O-acyltransferase 2; *APOF *apolipoprotein F; CYP27B1 cytochrome P450, subfamily XXVIIB, polypeptide 1; * LCAT *lecithin: cholesterol acyltransferase; *PLAGL1* pleomorphic adenoma gene-like 1; *ACOX2* acyl-CoA oxidase 2, *LIPC* lipase, hepatic; *ABCB10* ATP-binding cassette, subfamily B, member 10; *GGPS1* geranylgeranyl diphosphate synthase 1; *ACAT1* acetyl-CoA acetyltransferase 1; *APOA1* apolipoprotein A-I; *APOC3* apolipoprotein C-III; *CYP11A* cytochrome P450, subfamily XIA, polypeptide 1; *LRP3* low density lipoprotein receptor-related protein 3; *APOE* apolipoprotein E; *LIPE* lipase, hormone-sensitive; * APOC1* apolipoprotein C-I; *PPARG* peroxisome proliferator-activated receptor-gamma; *APOB* apolipoprotein B; *ABCG5* ATP-binding cassette, subfamily G, member 5; *ABCG8* ATP-binding cassette, subfamily G, member 8; *LDLR* low density lipoprotein receptor; *PCSK9* proprotein convertase, subtilisin/kexin-type, 9; *LDLRAP1* low density lipoprotein receptor adaptor protein 1; *CYP4A11* cytochrome P450, subfamily IVA, polypeptide 11; *FABP1* fatty acid-binding protein 1; *ABCC8* ATP-binding cassette, subfamily C, member 8; *LRP4* low density lipoprotein receptor-related protein 4; *ACAD8* acyl-CoA dehydrogenase family, member 8; *INSIG1* insulin-induced GENE 1; *ABCF2* ATP-binding cassette sub-family F member 2; *FABP5L3* fatty acid binding protein 5-LIKE 3; *INSR* insulin receptor; *C3* complement component 3.

**Table 2.** Candidate Genes in Relation to Coronary Atherosclerosis and Myocardial Infarction

Gene	OMIM gene ID	Chromosome	Substitution (SNP rs#)	subPSEC	P_deleterious_	Reference
*ALOX5AP*	603700	13q12	Y133H (rs41323349)	-0.31	0.06	[220]
*LTA4H*	151570	12q22	T600S (rs1803916)	-2.62	0.41	[220]
*BMPR1A*	601299	10q22.3	R443C (rs35619497)	-8.29	0.99	[211]
*LGALS2*	150571	22q13.1	A25P (rs9607476)	-1.86	0.24	[230]
			V119I (rs2235339)	-0.99	0.12	

*ALOX5AP* arachidonate 5-lipoxygenase-activating protein, *LTA4H* leukotriene A4 hydrolase, *BMPR1A *bone morphogenetic 	protein receptor type, *LGALS2* lectin galactoside-binding soluble 2, OMIM online mendelian inheritance in man, q long arm of a chromosome, SNP single nucleotide polymorphism, * http://www.ncbi.nlm.nih.gov/	(Genome build 36.1).
